# Fœtus in Utero, Killed by Lightning

**Published:** 1853-08

**Authors:** James Y. Carithers

**Affiliations:** Hendricksville, Alabama


From the Southern Medical and Surgical Journal.

*Fœtus in Utero, killed by Lightning.* By James Y. Carithers, M. D, of Hendricksville, Alabama.

Mrs. F——, aged about 40 years, in good health, and eight months advanced in pregnancy, received on the 10th of June, 1852, a severe shock from a streak of lightning, from which she recovered in a few hours—when she was attacked with labour pains which caused me to be sent for. On my arrival, I found her suffering with sharp pains. On examination, per vaginam, no dilatatation of the os uteri had taken place. Bled her freely, and ordered her an enema of a gill of starch, with a teaspoonful of laudanum, and to take ¼ of a gr. of sulph. morphine every half hour, until she was relieved from pain. After taking the fourth dose the pains subsided. Ordered her to take on the following morning ol. ricini, ℥i. At 2 P. M., oil acted freely on the bowels, and at 4, P.M., I found her resting well. Allowed some light nourishment, from that time until she was delivered, which took place on the tenth day after she complained of being very unwell. The child was dead, and from the appearance, had been so since the time the mother felt the shock.

